# Baseline neutrophil-to-lymphocyte ratio (NLR) and derived NLR could predict overall survival in patients with advanced melanoma treated with nivolumab

**DOI:** 10.1186/s40425-018-0383-1

**Published:** 2018-07-16

**Authors:** Mariaelena Capone, Diana Giannarelli, Domenico Mallardo, Gabriele Madonna, Lucia Festino, Antonio Maria Grimaldi, Vito Vanella, Ester Simeone, Miriam Paone, Giuseppe Palmieri, Ernesta Cavalcanti, Corrado Caracò, Paolo Antonio Ascierto

**Affiliations:** 1 Unit of Melanoma, Cancer Immunotherapy and Development Therapeutics, Istituto Nazionale Tumori IRCCS Fondazione “G. Pascale”, Via Mariano Semmola, 80131 Naples, Italy; 2 Statistical Unit, Regina Elena National Cancer Institute, Rome, Italy; 3 Unit of Cancer Genetics, Institute of Biomolecular Chemistry, CNR, I-07100 Sassari, Italy; 4 Department of Diagnostic Pathology and Laboratory, Istituto Nazionale Tumori- IRCCS –Fondazione G. Pascale, Napoli, Italy; 5 Melanoma and Skin Cancers Surgery Unit, Istituto Nazionale Tumori IRCCS Fondazione “G. Pascale”, Napoli, Italy

**Keywords:** PD-1 inhibitor, Nivolumab, Biomarkers, Neutrophil-to-lymphocyte ratio

## Abstract

**Background:**

Previous studies have suggested that elevated neutrophil-to-lymphocyte ratio (NLR) is prognostic for worse outcomes in patients with a variety of solid cancers, including those treated with immune checkpoint inhibitors.

**Methods:**

This was a retrospective analysis of 97 consecutive patients with stage IV melanoma who were treated with nivolumab. Baseline NLR and derived (d) NLR were calculated and, along with other characteristics, correlated with progression-free survival (PFS) and overall survival (OS) in univariate and multivariate analyses. The best cutoff values for NLR and dNLR were derived using Cutoff Finder software based on an R routine which optimized the significance of the split between Kaplan-Meier survival curves.

**Results:**

In univariate analysis, increasing absolute neutrophil count (ANC), NLR, dNLR and lactate dehydrogenase (LDH) (continuous variables) were all significantly associated with OS. Only NLR (hazard ratio [HR] = 2.85; 95% CI 1.60–5.08; *p* < 0.0001) and LDH (HR = 2.51; 95% CI 1.36–4.64; *p* < 0.0001) maintained a significant association with OS in multivariate analysis. Patients with baseline NLR ≥5 had significantly worse OS and PFS than patients with NLR < 5, as did patients with baseline dNLR ≥3 versus < 3. Optimal cut-off values were ≥ 4.7 for NLR and ≥ 3.8 for dNLR. Using this ≥4.7 cut-off for NLR, the values for OS and PFS were overlapping to the canonical cut-off for values, and dNLR< 3.8 was also associated with better OS and PFS.

**Conclusion:**

Both Neutrophil-to-lymphocyte ratio (NLR) and derived (d) NLR were associated with improved survival when baseline levels were lower than cut-off values. NLR and dNLR are simple, inexpensive and readily available biomarkers that could be used to help predict response to immunotherapy in patients with advanced melanoma.

**Electronic supplementary material:**

The online version of this article (10.1186/s40425-018-0383-1) contains supplementary material, which is available to authorized users.

## Background

Improved understanding of cancer and the role of the immune response has resulted in the development of new therapies, including immune checkpoint inhibitors targeting the cytotoxic T-lymphocyte-associated protein (CTLA)-4 (e.g. ipilimumab) and programmed death (PD)-1 receptors (e.g. nivolumab and pembrolizumab). These agents have revolutionized the treatment and outcomes of various cancers, in particular melanoma, with improved long-term disease control and prolonged patient survival [1, 2].

Nivolumab (Opdivo®, Bristol-Myers Squibb) is a fully human IgG4 monoclonal antibody that blocks the PD-1 receptor, a negative regulator of T-cell activity that has been shown to be involved in the control of T-cell immune responses. Engagement of PD-1 with its ligands PD-L1 or PD-L2 expressed on antigen-presenting cells and on tumor cells results in inhibition of T-cell proliferation and cytokine secretion, potentiating T-cell responses. Nivolumab has demonstrated clinical activity in previously treated and treatment-naïve patients with advanced melanoma, with overall response rates (ORR) ranging between 20 and 40% in patients treated at various dose levels [3–5]. Nevertheless, a significant portion of patients do not benefit from nivolumab treatment. Given that immunotherapy can be associated with significant toxicity as well as high treatment costs, it is important that those patients most likely to respond to treatment are better identified. To achieve this involves the recognition and validation of novel biomarkers predictive of treatment outcomes. Any such biomarkers should ideally be simple and easy-to-use, inexpensive and readily available.

In recent years, it has become evident that cancer-related inflammation responses, such as increased and defective myelopoiesis, and both local and systemic inflammation, have an important role in tumorigenesis, disease progression and patient prognosis [6, 7]. Therefore, predictive biomarkers that reflect the inflammatory response to treatment may help clinical decision making in the management of patients with melanoma. Systemic inflammation is associated with alterations in peripheral blood leukocytes that can be captured by the neutrophil-to-lymphocyte ratio (NLR) [8]. Several studies have demonstrated that NLR predicts outcomes in patients with a variety of solid cancers [9, 10]. In melanoma, raised leukocyte, neutrophil and monocyte counts, as well as high NLR have been associated with poor prognosis in patients with advanced melanoma, most of whom were receiving immunotherapies [11, 12]. Furthermore, in a recent Italian study, elevated NLR and derived (d) NLR were independently and significantly associated with an increased risk of death and disease progression in melanoma patients receiving ipilimumab [13].

This retrospective study investigates the role of the NLR and dNLR as predictive markers of response to treatment with nivolumab in patients with advanced melanoma.

## Methods

### Patients selection

A total of 97 consecutive patients with stage IV melanoma who were treated with nivolumab 3 mg/kg in a 60-min intravenous (IV) infusion every 2 weeks at the NCI Fondazione “G. Pascale” of Napoli between February 2015 and February 2017 had available hematological values and were included in this analysis. For all patients, clinical data (white blood cell [WBC] count, absolute lymphocyte count [ALC], absolute neutrophil count [ANC], serum lactate dehydrogenase [LDH], and other clinical data, including BRAF status, brain metastasis, lines of prior treatment, and response) were collected before starting nivolumab treatment and until last follow-up. Patients were treated with nivolumab until disease progression, intolerable toxicity and/or the investigator’s decision. Tumor assessment was performed at baseline, at week 12, and every 12 weeks thereafter, and clinical response were classified according to response evaluation criteria in solid tumors (RECIST) [14].

### Statistical analysis

Descriptive statistics were used to summarize patient and treatment characteristics. NLR was calculated as NLR = ANC/ALC and dNLR was calculated as dNLR = ANC/(WBC − ALC). Progression-free survival (PFS) was determined from the first cycle of treatment to disease progression documented by imaging, or death (event), or last follow-up (censored). Overall survival (OS) was calculated from the first cycle of treatment to death (event) or last follow-up (censored). Multivariate Cox proportional hazard models were used to investigate associations of NLR with survival, adjusted for baseline characteristics. Results were presented as hazard ratios (HR) with 95% confidence intervals (CIs). The best cutoff was derived using Cutoff Finder software [15] based on an R routine which optimized the significance of the split between Kaplan-Meier survival curves measured by the log-rank test. Statistical analyses were performed with IBM-SPSS version 21.0.

## Results

Baseline patient characteristics are summarized in Table 1. The median age at first treatment was 61 years (range 21–85) and 56.7% of patients were male. A total of 27 patients (27.8%) had CNS metastases. BRAF-mutated melanomas were present in 38 patients (39.1%); 54 (55.7%) had BRAF wild-type tumors. Twenty-five patients (25.8%) had received one previous line of therapy, 48 (49.5%) had two lines and 24 (24.7%) had ≥3 lines.

**Table 1:** Baseline patient characteristics

	*N* = 97
Gender, *N* (%), Female/Male	42 (43.2) / 55 (56.7)
Age, years, median (range)	61 (21–85)
Number of previous therapies, *N* (%)	
1	25 (25.8)
2	48 (49.5)
≥ 3	24 (24.7)
Central nervous system (CNS) metastasis	27 (27.8)
Baseline lactate dehydrogenase, *N* (%)	
≤ upper limit of normal (ULN)	50 (51.5)
> ULN	45 (46.4)
Unknown	2 (2.1)
B-RAF, *N* (%):	
Mutation	38 (39.1)
Wild-Type	54 (55.7)
Unknown	5 (5.2)

As of May 2017, 44 patients (45.4%) were still alive. The median PFS was 4 months (range 0–26) and median OS was 6 months (range 2–26); about 10% of patients had started therapy no more than 6 months earlier. There was no significant difference in OS by BRAF status (*P* = 0.65). Overall, 57 patients (58.8%) had progressive disease (PD), 20 patients (20.6%) had a partial response (PR) and 20 patients (20.6%) had stable disease (SD). The disease control rate (DCR) was 41.2%.

At baseline, median WBC was 8620/μL cells in patients who had PD, 7540/μL in patients with SD and 6645/μL in patients with PR (median 7.000/μL in patients with disease control). A total of 22 patients (22.7%) had neutrophilia (ANC > 7.5) before the start of treatment.

### ANC

In univariate analysis, elevated ANC (> 8000) was associated with poor prognosis, with a median of OS of 2.6 months compared to 16.0 months in patients with normal ANC (*P* < 0.0001).

Using median ANC as cut-off value, median OS was 16 months (95% CI: 7.6–24.4) in patients with ANC < 5.4 compared to 5.7 months (95% CI: 2.6–8.8) in patients with ANC ≥5.4 (HR 2.04; 95% CI: 1.17–3.57; *P* = 0.01). Median PFS was also increased in patients with ANC < 5.4 compared with ANC ≥5.4 (8 months [95% CI: 1.6–14.4] versus 3 months [95% CI:1.5–4.5]) but this was not statistically significant (HR 1.60; 95% CI: 0.98–2.61; *P* = 0.06) (See Additional files 1 and 2).

### NLR and dNLR

At baseline, 27 (27.8%) patients had a NLR ≥5 and 31 (32%) patients had a dNLR ≥3, the cut-offs most often indicated in the literature. Using Harrell’s c-index to determine the prognostic power of NLR and dNLR, we obtained a good prognostic value for both, with c = 0.72 (95% CI: 0.67–0.77) for NLR and c = 0.70 (95% CI: 0.65–0.75) for dNLR.

#### PFS

**Fig. 1:**
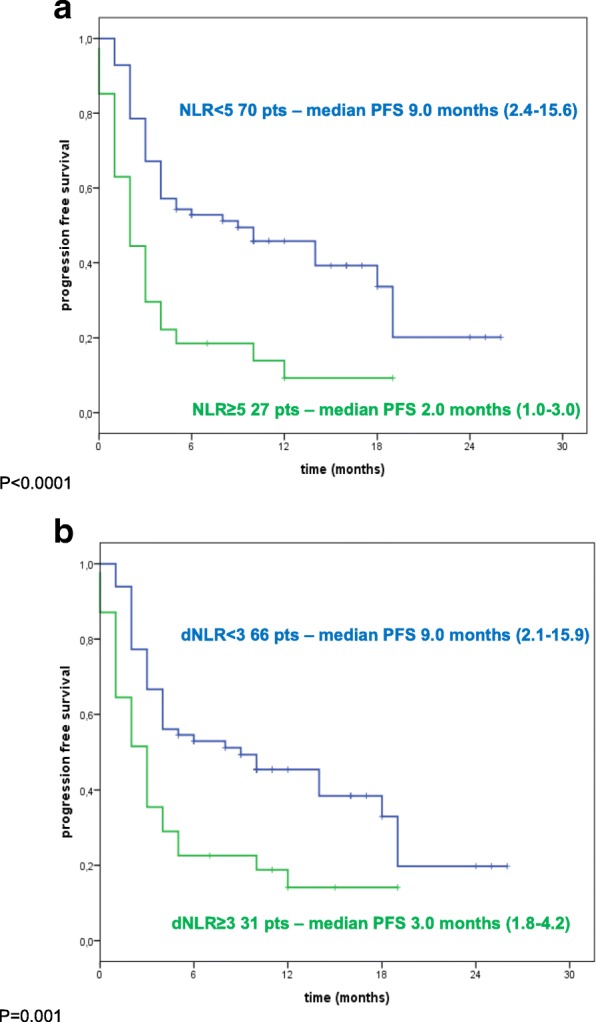
Kaplan-Maier PFS curves of melanoma patient treated with nivolumab. **a** Patients stratified according baseline neutrophils-to lymphocyte ratio (NLR). Green line: NLR ≥ 5; Blue line: NLR < 5. **b** Patients stratified according baseline derived neutrophils-to lymphocyte ratio (dNLR). Green line: dNLR≥3; Blue line: dNLR< 3

When a baseline NLR value of 5 was used as the cut-off, patients with NLR < 5 (*n* = 70; 72.2%;) had a significantly longer median PFS of 9 months (95% CI: 2.4–15.6) compared to patients with NLR ≥5 (median PFS of 2 months, 95% CI: 1.0–3.0; *P* < 0.0001) (Fig. 1a). Patients with baseline dNLR < 3 (*n* = 66; 68%) had a median PFS of 9 months (95% CI: 2.1–15.9) while patients with dNLR ≥3 (*n* = 31; 32%) had a median PFS of 3 months (95% CI: 1.8–4.2) (*P* = 0.001) (Fig. 1b).

#### OS

**Fig. 2:**
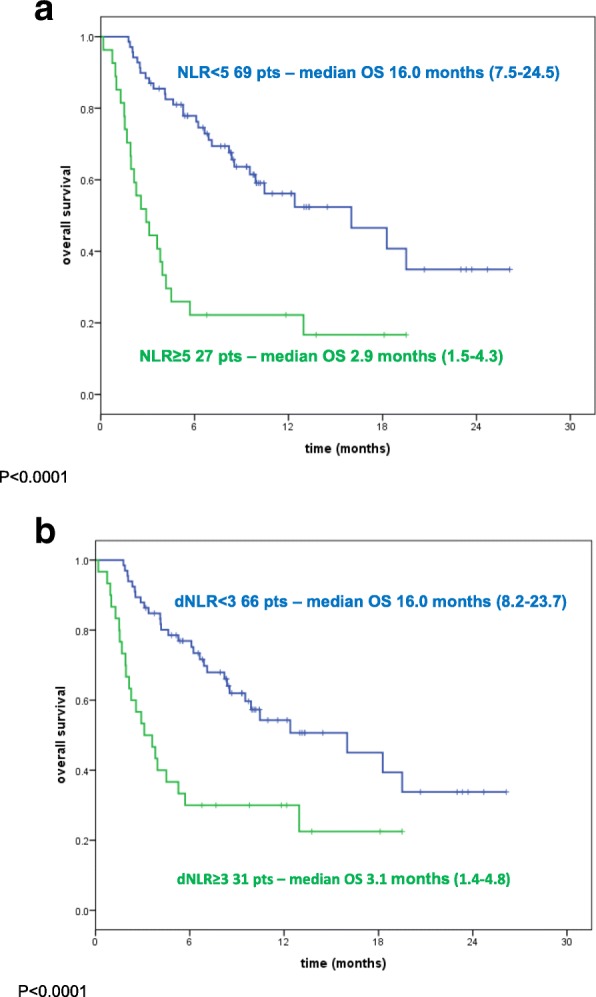
Kaplan-Maier overall survival curves of melanoma patient treated with nivolumab. **a** Patients stratified according baseline neutrophils-to lymphocyte ratio (NLR). Green line: NLR ≥ 5; Blue line: NLR < 5. **b** Patients stratified according baseline derived neutrophils-to lymphocyte ratio (dNLR). Green line: dNLR≥3; Blue line: dNLR< 3

Median OS was 2.9 months (95% CI: 1.5–4.3) for patients with baseline NLR ≥5 compared with 16 months for patients with NLR < 5 (95% CI: 7.5–24.5) (*P* < 0.0001) *(*Fig. 2a). Using dNLR ≥3 as cut-off the difference was significant, with a median OS of 16.0 months (95% CI: 8.2–23.7) for patients with dNLR < 3 and 3.1 months (95% CI: 1.4–4.8) for patients with dNLR ≥3 (*P* < 0.0001) (Fig. 2b).

#### Response

The proportion of patients with an NLR ≥5 was 20% (4/20) in patients with a PR, 5% (1/20) in patients with with SD and 38.6% (22/57) in patients with PD.

In order to better investigate the role of NLR and dNLR, the optimal cutoffs were derived by maximizing the significance assessed by the log-rank test; these values were 4.7 for NLR and 3.8 for dNLR. The performance of an NLR cut-off value of 4.7 was overlapping with 5 as cut-off value, Median PFS and OS were also significantly longer in patients with dNLR < 3.8 compared to ≥3.8 (see Additional files 1 and 2).

In the subgroup of 27 patients with brain metastases, NLR < 4.7 and dNLR < 3.8 were both significantly associated with improved OS and PFS in univariate analysis. However, in multivariate analysis, only NLR < 4.7 was associated with significantly longer OS (HR 3.22, 95% CI: 0.99–10.51) and PFS (HR 2.55, 95% CI: 0.98–6.65) (See Additional files 1 and 2).

Univariate analysis using a Cox model was performed using median values as cut-off for WBC, ANC, and LDH; these three variables together with NLR and dNLR were all significantly associated with OS, while there was no significant association with sex, age, number of lines of treatment, presence of brain metastases or BRAF mutation status. In multivariate analysis, only NLR (HR = 2.85; 95% CI: 1.60–5.08; *P* < 0.0001) and LDH (HR = 2.51; 95% CI: 1.36–4.64; *P* < 0.0001) maintained a significant association with OS (Table 2). When considering PFS, LDH, NLR and dNLR showed a prognostic role in univariate analysis while, in multivariate analysis, associations were only observed for NLR (HR = 2.10; 95% CI: 1.23–3.59; *P* = 0.007) and LDH (HR = 1.74; 95% CI: 1.03–2.94; *P* = 0.04) (Table 2).

**Table 2:** Cox regression models on overall survival and progression-free survival

	Overall survival		Progression-free survival	
Factors	Univariate HR, 95% CI, *P*-value	Multivariate HR, 95% CI, *P*-value	Univariate HR, 95% CI, *P*-value	Multivariate HR, 95% CI, *P*-value
Sex (M vs F)	1.71 (0.96–3.05), *P* = 0.07		1.35 (0.82–2.21) *P* = 0.24	
Age (years)	1.00 (0.98–1.02), *P* = 0.72		1.00 (0.98–1.01) *P* = 0.74	
Lines of treatment:	*P* = 0.55		*P* = 0.63	
2 vs 1	1.23 (0.57–2.64)		1.06 (0.56–1.99)	
≥ 3 vs 1	1.56 (0.69–3.55)		1.35 (0.67–2.73)	
BRAF (mutant vs wild-type)	1.14 (0.64–2.04), *P* = 0.65		1.32 (0.80–2.19) *P* = 0.28	
Brain metastases (yes vs no)	1.03 (0.56–1.87), *P* = 0.93		1.30 (0.77–2.18) *P* = 0.32	
Lactate dehydrogenase (≥454 vs < 454)	3.06 (1.69–5.57), *P* < 0.0001	2.51 (1.36–4.64), *P* = 0.003	2.16 (1.18–3.99) *P* = 0.01	1.74 (1.03–2.94) *P* = 0.04
Absolute neutrophil count (≥5.4 vs < 5.4)	2.04 (1.17–3.57), *P* = 0.01		1.60 (0.98–2.61) *P* = 0.06	
White blood cell count (≥7.6 vs < 7.6)	1.57 (0.91–2.73), *P* = 0.11		1.55 (0.95–2.54) *P* = 0.08	
Neutrophil-to-lymphocyte ratio (≥5.0 vs < 5.0)	3.53 (2.02–6.16), *P* < 0.0001	2.85 (1.60–5.08), *P* < 0.0001	2.53 (1.53–4.18) *P* < 0.0001	2.10 (1.23–3.59) *P* = 0.007
Derived neutrophil-to-lymphocyte ratio (≥3.0 vs < 3.0)	2.70 (1.55–4.69), *P* < 0.0001		2.50 (1.48–4.23) *P* = 0.001	

**Fig. 3:**
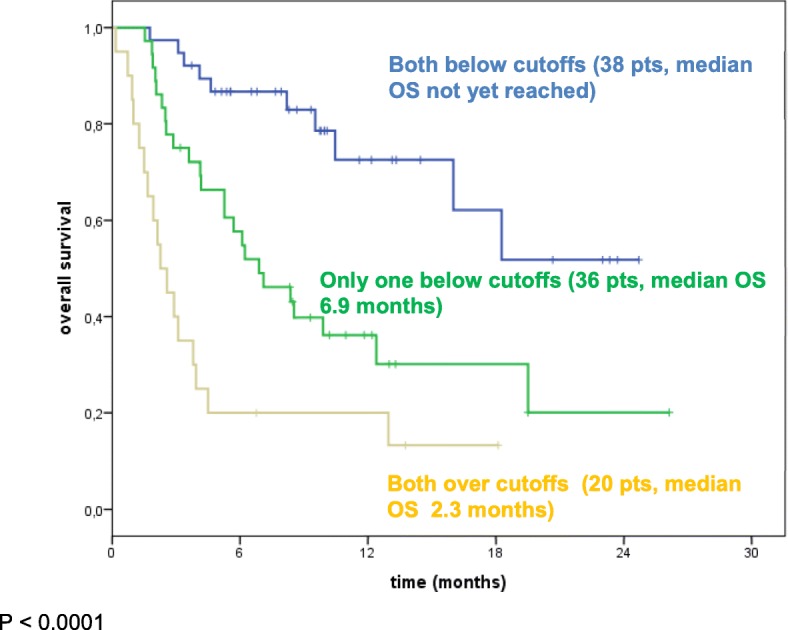
Kaplan-Maier OS curves of melanoma patients treated with nivolumab and stratified according to LDH and NLR (LDH cutoff = 455 mL/U; NLR cutoff = 5). Green line: only one factor below cutoffs; Yellow line: both factors over cutoffs; Blue line: Both factors below cutoffs

These two factors can be used to stratify patients into three groups: both elevated NLR (≥5.0) and elevated LDH (≥455 mL/U) with a median OS of 2.3 months (95% CI: 1.3–3.2); either elevated NLR (≥5.0) or elevated LDH (≥455 mL/U) with a median OS of 6.9 months (95% CI: 3.9–9.9) and normal NLR and LDH (median OS is not yet reached, 1-year OS 72.5%) (Fig. 3).

## Discussion

Melanoma is an aggressive cancer with frequent relapse and a high potential for metastasis. Nevertheless, in recent years, the range of therapeutic options for melanoma has been greatly improved with the introduction of immunomodulatory antibodies as well as molecular targeted drugs [16]. These agents have shown promising results, but not all patients benefit and toxicity can be a significant concern. Because of this, it is important to be able to identify which patients are most likely to benefit from treatment.

In recent years, NLR has been extensively studied as a prognostic marker in patients with solid tumors on the basis that it reflects the inflammatory response to cancer. In patients with advanced cancer, there is usually a change in peripheral blood cell composition that is suggestive of an expansion in the myeloid component (neutrophils and monocytes) and a reduction in the lymphoid compartment.

A number of studies have shown that systemic inflammation markers are associated with the outcome of malignant melanoma patients, including those treated with ipilimumab [13, 17, 18]. In 69 patients with metastatic melanoma, pre-treatment NLR was identified as an independent marker for response in multivariate analyses, with patients with baseline NLR ≥5 having significantly worse PFS and OS compared with those with a NLR < 5 [18]. Similarly, a significant association between elevated NLR and worse survival was significant after adjustment for potential confounders in an analysis of 58 patients treated with ipilimumab [19]. In another study of patients with unresectable stage III-IV melanoma, elevated NLR (≥5) was associated with worse OS, PFS, and clinical response [20]. In addition, increasing NLR from baseline during treatment was correlated with worse OS and PFS in patients treated with ipilimumab but not those treated with vemurafenib, suggestive of a predictive value for response to immunotherapy but not targeted drugs. In another recent study, in 162 melanoma patients, 22.5% receiving ipilimumab, elevated baseline dNLR (≥3) was independently and significantly associated with an increased risk of death and disease progression [13].

Elevated baseline NLR ≥ 5 has also been associated with worse outcomes in patients treated with nivolumab, although this was in non-small-cell lung cancer (NSCLC) rather than melanoma [21]. In a retrospective analysis of 175 patients with previously treated advanced NSCLC, NLR ≥5 was independently associated with reduced OS (median 5.5 vs. 8.4 months; HR 2.07, 95% CI: 1.3–3.3; *p* = 0.002) and PFS (median 1.9 vs. 2.8 months; HR 1.43, 95% CI: 1.02–2.0; *p* = 0.04). Data from the Italian nivolumab extended access program (EAP) has also reported that NLR (≥3) was a prognostic factor for OS in patients with metastatic renal cell cancer treated with nivolumab [22].

In this study, which is the first to report an association between NLR and outcomes in patients with advanced melanoma treated with nivolumab, we analyzed the predictive power of baseline NLR and dNLR in a small retrospective cohort of patients. Despite the small number of patients, both NLR and dNLR were good predictors of survival when baseline levels were lower than cut-off values. The optimal cut-off values were shown to be ≥4.7 for NLR and ≥ 3.8 for dNLR, with the NLR cut-off value a more robust predictor than the dNLR value. NLR was predictive of survival independent of BRAF status (data not shown). Lower baseline LDH also correlated with a better response and survival, consistent with several previous studies [23–25], which was maintained in multivariate analysis. Patients with two or three risk factors of elevated NLR, ANC and LDH had worse survival than patients with none or just one of these factors alone. These results suggest that the systemic inflammatory response is a potent stimulator of cancer progression in established disease. Elevated NLR suggests a systemic inflammatory state and can be indicative of neutrophilia, lymphopenia, or a combination of both.

The biggest limitation of this study is the low number of patients that we have analyzed and validation is required in larger prospective studies. The benefits that can be gained with the validation of these parameters may be significant because they offer the potential for a low-cost, non-invasive and easy-to-read test that can be used to help define the patient’s status, prognosis, and response to therapeutic treatment.

## Conclusion

Neutrophilia can occur in cancer patients at a peripheral level, but neutrophils can also localize to the tumor due to multiple factors, including general inflammatory signals such as IL-1 and TNF-α [26]. Although many mechanisms are unclear, neutrophils are thought to primarily propagate a pro-tumor environment promoting angiogenesis and tumor growth, degrading the extracellular matrix and providing favorable conditions for metastasis, and potentiating genome instability and tumor evolution [27]. A recent study also demonstrated that the expression of PD-L1 on tumor infiltrating neutrophils could functionally inhibit the activation of T cells [28]. Lymphopenia in the context of cancer also suggests more aggressive disease progression. For these reasons, whether due to neutrophilia or lymphopenia, elevated NLR physiologically suggests an inability of the immune system to suppress cancer progression, and thus it can be an index for OS and PFS. Because the NLR and dNLR are obtained from complete blood counts, their use does not involve additional procedures for patients or extra resources and associated costs for healthcare providers. If it can be shown that NLR and dNLR are predictors of response to immunotherapy as well as being prognostic markers, their use in clinical practice could allow better patient selection and optimal clinical management.

## Additional files

Additional file 1: **Table S1A.** Cox regression models on overall survival in patients with brain metastases (DOCX 15 kb)

Additional file 2: **Figure S1.** Kaplan-Maier OS and PFS curves of melanoma patient treated with nivolumab. (A) Patients stratified according baseline median ANC as cutoff . Green line: ANC≥5.4; Blue line: ANC<5.4. (B) Patients stratified for PFS according baseline median ANC as cut-off . Green line: ANC≥5.4; Blue line: ANC<5.4. **Figure S2.** Kaplan-Maier OS and PFS curves of melanoma patient treated with nivolumab using optimal cutoff for derived neutrophils-to lymphocyte ratio (dNLR). (A)) Patients stratified according baseline dNLR. Green line: dNLR≥3.8; Blue line: dNLR<3.8. (DOCX 168 kb)
