# Supplementary material for: Baseline neutrophil-to-lymphocyte ratio (NLR) and derived NLR could predict overall survival in patients with advanced melanoma treated with nivolumab
Source: J Immunother Cancer. 2018 Jul 16;6:74. doi: 10.1186/s40425-018-0383-1 (PMC6048712; doi:10.1186/s40425-018-0383-1)
Supplement: Supplementary file 1 — Table S1A. Cox regression models on overall survival in patients with brain metastases (DOCX 15 kb) [file 40425_2018_383_MOESM1_ESM.docx]

**Supplementary Table**

**Table 1**

| **Factors** | **Univariate HR, 95%c.i., P value** | **Multivariate HR, 95%c.i., P value** |
| --- | --- | --- |
| Sex (m vs f) | 1.31 (0.44-3.90) P=0.62 |  |
| Age (years) | 1.04 (1.00-1.08) P=0.04 | 1.06 (1.01-1.10) P=0.01 |
| Line of treatment  2 vs 1  >=3 vs 1 | P=0.43  1.53 (0.33-7.21)  0.71 (0.13-4.03) |  |
| BRAF (mut vs wt) | 0.85 (0.29-2.49) P=0.77 |  |
| LDH (>454 vs <454) | 1.88 (0.63-5.60) P=0.26 |  |
| ANC (> 5.4 vs < 5.4) | 2.42 (0.82-7.12) P=0.11 |  |
| WBC (>7.6 vs < 7.6) | 1.61 (0.51-5.10) P=0.41 |  |
| NLR (>4.7 vs < 4.7) | 3.27 (1.11-9.61) P=0.03 | 3.22 (0.99-10.51) P=0.05 |
| dNLR (>3.8 vs < 3.8) | 3.27 (1.11-9.61) P=0.03 |  |

**Table 1A**.**Cox regression models on overall survival in patients with brain metastases**

| **Factors** | **Univariate HR, 95%c.i., P value** | **Multivariate HR, 95%c.i., P value** |
| --- | --- | --- |
| Sex (m vs f) | 0.95 (0.39-2.33) P=0.91 |  |
| Age (years) | 1.02 (0.98-1.05) P=0.41 |  |
| Line of treatment  2 vs 1  >=3 vs 1 | P=0.35  0.99 (0.31-3.15)  0.46 (0.12-1.80) |  |
| BRAF (mut vs wt) | 1.13 (0.44-2.90) P=0.79 |  |
| LDH (>454 vs <454) | 1.29 (0.53-3.14) P=0.58 |  |
| ANC (> 5.4 vs < 5.4) | 2.53 (0.99-6.42) P=0.05 |  |
| WBC (>7.6 vs < 7.6) | 2.08 (0.75-5.76) P=0.16 |  |
| NLR (>4.7 vs < 4.7) | 2.55 (0.99-6.65) P=0.05 | 2.55 (0.98-6.65) P=0.05 |
| dNLR (>3.8 vs < 3.8) | 2.55 (0.99-6.65) P=0.05 |  |

**Table 1B. Cox regression models on progression free survival in patients with brain metastases**
