# Supplementary material for: Baseline neutrophil-to-lymphocyte ratio (NLR) and derived NLR could predict overall survival in patients with advanced melanoma treated with nivolumab
Source: J Immunother Cancer. 2018 Jul 16;6:74. doi: 10.1186/s40425-018-0383-1 (PMC6048712; doi:10.1186/s40425-018-0383-1)
Supplement: Supplementary file 2 — Figure S1. Kaplan-Maier OS and PFS curves of melanoma patient treated with nivolumab. (A) Patients stratified according baseline median ANC as cutoff . Green line: ANC≥5.4; Blue line: ANC<5.4. (B) Patients stratified for PFS according baseline median ANC as cut-off . Green line: ANC≥5.4; Blue line: ANC<5.4. Figure S2. Kaplan-Maier OS and PFS curves of melanoma patient treated with nivolumab using optimal cutoff for derived neutrophils-to lymphocyte ratio (dNLR). (A)) Patients stratified according baseline dNLR. Green line: dNLR≥3.8; Blue line: dNLR<3.8. (DOCX 168 kb) [file 40425_2018_383_MOESM2_ESM.docx]

**Supplementary Figures**

**Figure 1.** Kaplan-Maier OS and PFS curves of melanoma patient treated with nivolumab. (A) Patients stratified according baseline median ANC as cutoff . Green line: ANC≥5.4; Blue line: ANC<5.4. (B) Patients stratified for PFS according baseline median ANC as cut-off . Green line: ANC≥5.4; Blue line: ANC<5.4.

**Figure 1A**


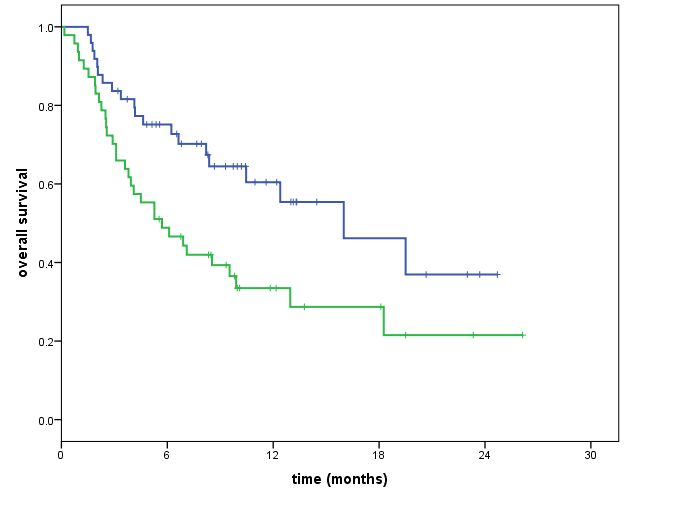


ANC < 5.4 median 16 months (95% c.i. 7.6-24.4)

ANC ≥ 5.4 median 5.7 months (95% c.i. 2.6-8.8)

P=0.01

**Figure 1B**


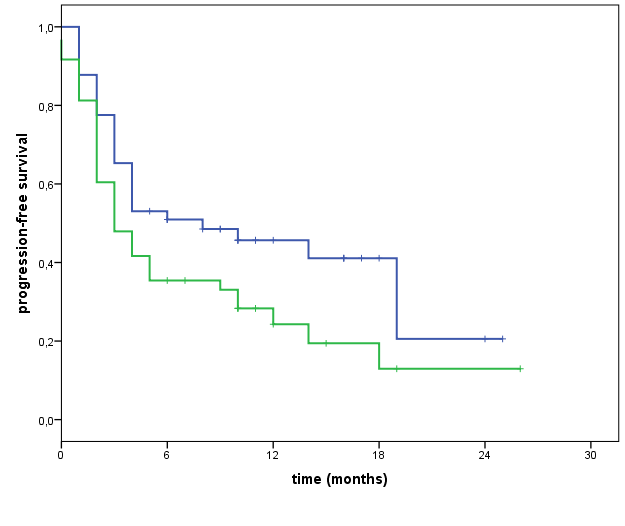


ANC < 5.4 median 8 months (95% c.i. 1.6-14.4)

ANC≥ 5.4 median 3 months (95% c.i. 1.5-4.5)

P=0.04

**Figure 2.** Kaplan-Maier OS and PFS curves of melanoma patient treated with nivolumab using optimal cutoff for derived neutrophils-to lymphocyte ratio (dNLR). (A) ) Patients stratified according baseline dNLR. Green line: dNLR≥3.8; Blue line: dNLR<3.8.


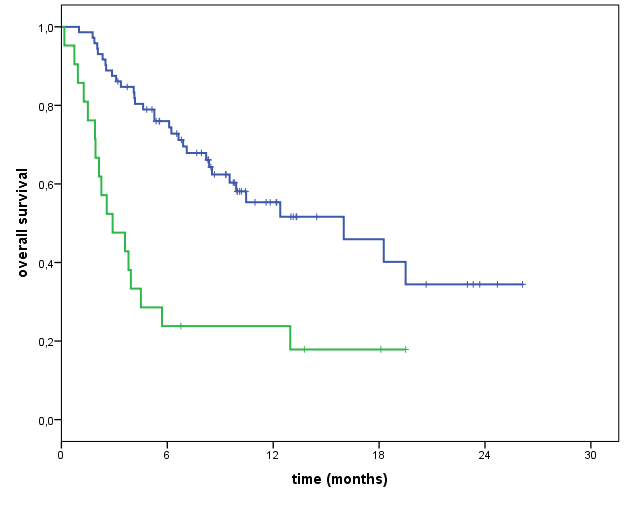


P<0.0001

**dNLR<3.8 73 pts – median OS 16.0 months (7.6-24.4)**

**dNLR>3.8 24 pts – median OS 2.9 months (0.9-4.9)**

**Figure 2A**

**Figure 2B**


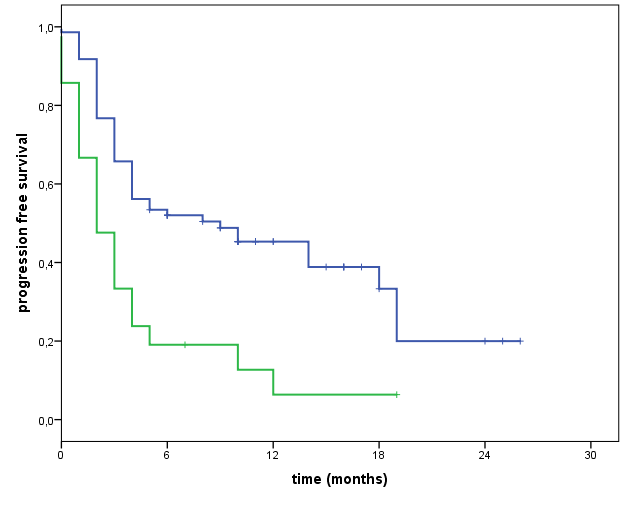


P<0.0001

**dNLR<3.8 73 pts – median PFS 9.0 months (2.3-15.7)**

**dNLR>3.8 23 pts – median PFS 2.0 months (0.8-3.2)**
